# Laws, Policies, and Collective Agreements Protecting Low-wage and Digital Platform Workers During the COVID-19 Pandemic

**DOI:** 10.1177/10482911221133796

**Published:** 2022-10-19

**Authors:** Ellen MacEachen, Angelique de Rijk, Johnny Dyreborg, Jean-Baptiste Fassier, Michael Fletcher, Pamela Hopwood, Meri Koivusalo, Shannon Majowicz, Samantha Meyer, Christian Ståhl, Felix Welti

**Affiliations:** 1 8430 University of Waterloo, Waterloo, Ontario, Canada; 2 5211 Maastricht University, Maastricht, Limburg, The Netherlands; 3 2686 National Research Centre for the Working Environment, København, Denmark; 4 Hospices Civils de Lyon, Lyon, Auvergne-Rhône-Alpes, France; 5 University of Sherbrooke, Sherbrooke, Quebec, Canada; 6 8491 Victoria University of Wellington, Wellington, New Zealand; 7 7840 Tampere University, Tampere, Pirkanmaa, Finland; 8 4566 Linköping University, Linkoping, Östergötland, Sweden; 9 9178 University of Kassel, Kassel, Hessen, Germany

**Keywords:** social security policy, digital platform gig work, self-employment, low wage, occupational health

## Abstract

In the context of the COVID-19 pandemic, this commentary describes and compares shifting employment and occupational health social protections of low-wage workers, including self-employed digital platform workers. Through a focus on eight advanced economy countries, this paper identifies how employment misclassification and definitions of employees were handled in law and policy. Debates about minimum wage and occupational health and safety standards as they relate to worker well-being are considered. Finally, we discuss promising changes introduced during the COVID-19 pandemic that protect the health of low-wage and self-employed workers. Overall, we describe an ongoing “haves” and a “have not” divide, with on the one extreme, traditional job arrangements with good work-and-health social protections and, on the other extreme, low-wage and self-employed digital platform workers who are mostly left out of schemes. However, during the pandemic small and often temporary gains occurred and are discussed.

## Introduction

In the context of COVID-19 and employment systems that have increasingly shifted workers into “good” jobs with benefits and “bad” dead-end jobs in neoliberal economies,^1^ what has been called “a new class divide” separated out “the remotes,” largely knowledge workers who could work from home, from “the essentials,” who were low-wage front-line service workers.^2^ Among the “essentials” were workers in low-wage jobs, such as store clerks, as well as digital platform couriers who continued to crisscross cities during lockdowns, for instance, to deliver meals and packages to factories, hospitals, and residences. Another relevant category of worker during the pandemic has been the “un/under employed.”^3,4^ These were the large number of workers, mostly in lower-paying jobs and often women or racialized persons, who lost their jobs or suffered lowered incomes during the pandemic. ^5–7^

Altogether, low-wage workers, including digital platform workers (sometimes called “gig workers”) who are mostly classified as self-employed, share similar job characteristics that placed them at particular risk during the pandemic. For instance, both groups face contract renewal uncertainty and income inadequacy or volatility.^1,8,9^ As well, self-employed workers are often left out of legal rights and protections such as the right to refuse unsafe work,^10^ while low-wage workers can be fearful about speaking up about unsafe work conditions due to uneven power relationships.^11,12^ As a result of pandemic economic slowdowns, many low-wage and platform workers lost employment. When they remained employed, their jobs often required continuous exposure to the public and the COVID-19 virus.^7,13,14^

Of concern is that, in many jurisdictions, social security systems have not fully protected injured workers who are precariously employed, including multiple job-holders and those with part-time jobs, zero-hours contracts, or classified as self-employed.^15–17^ Self-employed people are largely not covered by health and employment standards and most precariously employed workers are not covered by collective agreements; altogether, they have little negotiation power.^18,19^ For this reason, the European Union (EU) proposed a directive for collective agreements for “solo-self-employed” in line with the European Pillar of Social Rights, which was introduced in 2017^20^ and builds on the concept of “decent work” first coined by the International Labour Organization in 1999.^21^ As noted in the Organization for Economic Co-operation and Development (OECD) report on the future of work, low-skilled people are falling behind in the labor market and many of these changes appear to be structural rather than related to economic fluctuations.^22^

Digital platform economy expansion was accelerated by the rapid growth of e-commerce during the COVID-19 crisis.^23–25^ For instance, across OECD countries, self-employment rates range from approximately 9% in Canada, an average of 15% in EU, and 19% in New Zealand.^26^ However, these rates do not adequately capture digital platform workers, many of whom, as self-employed workers, are not fully engaged with their country's taxation systems. ^4,27^ The OECD defines platform work as activities that have in common the use of online platforms to connect the demand and supply of particular services including services performed digitally (e.g., clerical and data entry, translation, or design services) or services performed on location (i.e., transport, delivery, housekeeping).^16^

The aim of this commentary is to describe and compare key employment, work and health protections of low-wage and self-employed workers during the COVID-19 pandemic, up to early Wave 4 (September 2021). Specifically, we examine how, across advanced economy countries, laws, policies, and collective agreements did or did not protect the health of low-wage (e.g., service workers) and digital platform workers (usually classified as self-employed) including during the first three waves (2019–2021) of the COVID-19 pandemic. In the discussion, we consider current strategies to improve the working conditions of low-wage and digital platform workers by altering the employer role, state social security systems, or devising a new type of worker role.

We approach this issue via a broad lens of social contract for advanced industrial economies spanning Europe, Canada, and New Zealand. That is, we refer to common understandings of fair ways for distributing power and resources.^28^ Our overall goal is to inspire conversation, comment, critique, and new research questions to tackle the issue of the employment, work and health of low-wage workers and self-employed digital platform workers.

## Methods

This paper is undertaken by an international working group of researchers with practice and policy expertise in employment, work and health practice and policy. Disciplines in our working group span law, medicine, epidemiology, and social sciences. The 11 members of the group (all authors) were selected for their detailed knowledge, not only of policies, but also how these are applied within their jurisdictions of Sweden, Finland, Denmark, the Netherlands, France, Germany, New Zealand, and Canada. Six of these countries are member states of the EU and therefore governed by common EU regulations. As well, the European countries have more nationally defined regulations and laws in the field of social and health policy. These high-income countries were selected to provide a comparative analysis of employment and occupational health policy schemes across welfare states with long-standing membership in the Organisation for Economic Co-operation and Development.

In Spring 2021, members of the working group shared written responses to the following two questions:

- How do laws, policies, and practices (including collective agreements) in your country protect the health of low-wage (e.g., retail workers) and digital platform workers (e.g., self-employed Uber drivers)?
- What occupational health and public health interventions are being taken or considered in your jurisdiction for these workers in light of health risks related to the COVID-19 pandemic?

Following these submissions, exchanges took place to elaborate and clarify content related to each country. In September 2021, the working group held a virtual Policy Round Table to elaborate, compare, contrast, and synthesize our written submissions. The goal of the group was to identify key examples of policy, documented herein, relevant to the employment and occupational health of low-wage and digital platform workers rather than to comprehensively document all system protections.

## Findings

In our analysis, we present key differences between our countries in order to draw out how laws, policies, and collective agreements protected the health and safety of low-wage and self-employed digital platform workers during COVID-19 and insights for research and policy reform. The findings are presented in four parts. First, to set the stage for employment and occupational health protections for digital platform workers, we delineate efforts to address employment misclassification across the countries. Second, we describe the changing landscape for key standards in place for income security and occupational health and safety protections. Then, we describe differences between protections and benefits available to employees relative to non-employees with respect to key policy areas designed to keep workers healthy and secure: unemployment benefits and sickness and injury benefits. Finally, we discuss how changes introduced during the COVID-19 pandemic protected the health and safety of low-wage and self-employed workers. Our discussion centers on areas for policy reform and research.

1. *Employment classification*

Employee status is almost always key to workers accessing important social protections and benefits. In all eight jurisdictions addressed by this working group, digital platform workers have been largely classified as self-employed. In some jurisdictions, platform workers have been allocated to a third employment category that provides some basic rights (e.g., minimum wage) but not full employee rights. An example is the “worker” category newly created in the UK in 2021 for Uber drivers.^29^ While some platform workers are genuinely self-employed and autonomous in their work, other platform workers are misclassified as self-employed (or as a third category) and are excluded from full employment protections, even though many platforms have considerable control over the pay and conditions of work. ^25,30–32^ Employment misclassification is also a growing challenge in other sectors, including meat packing and parcel delivery.^33–35^ By employment misclassification, we mean attempts to disguise the employment relationship that have the effect of depriving workers of the social protections that they are due.^36^ Misclassification practices exclude increasing numbers of workers from large portions of our employment standards and social safety nets,^37^ such as minimum wage standards, unemployment income benefits, and workers’ compensation coverage.

To date, some efforts have been made to require employers, rather than workers, to prove that workers should be classified as self-employed. For instance, in Canada, the Ontario government introduced a bill in 2017 that placed the onus on employers to prove their independent contractors were not “employees.” However, this was reversed when a subsequent Conservative government returned the onus to independent contractors (who are often economically insecure, lacking legal support funds) to prove they are actually employees. Like the 2017 effort in Ontario, a December 2021 European Commission drafted a directive to improve digital labor platform working conditions that proposes that workers are presumed to be employees—this places the onus on employers to prove otherwise. This directive proposes that if the rebuttal of this presumption rule takes a long time to determine in court cases, then the worker will have the employment rights in the meantime.^38^ EU directives are unlike regulations in that they give Member States more leeway in how aims of the directive are achieved. While this directive sets out a goal that all EU countries must achieve, it is up to the individual countries to devise their own laws on how to reach these goals.^39^ The directive is likely to become law, but changes to the proposal might occur after the hearing process and before the directive is concluded. It is likely that the proposed directive will have different implications for different types of platform workers. Following confirmation of the final version of the directive, Member States are expected to transpose this into national legislation or collective agreements.

New Zealand is also actively working to address employment misclassification by clarifying the legal definitions of employee. In December 2021, a Tripartite Working Group of unions, employers and government-recommended law reform to address the employee/contractor boundary issue. The recommended reform included revising the legislative definition of “employee” to include a contradistinction to someone who is genuinely in business on their own account and to include more detailed, objective and prescriptive legislative requirements for worker classification.^40^ The New Zealand government has not yet implemented these recommendations.

Other efforts to address misclassification have been to ban subcontracting in a vulnerable sector, and organized labor campaigns. For instance, Germany created legislation in 2020 that banned subcontracts in the slaughterhouse sector, following findings of employment misclassification and poor working conditions among workers who were largely recent immigrants.^33^ It should be noted that subcontracts were not banned in sectors where the workers are legally classified as independent.

An interesting exception to the exclusion of self-employed workers from employment protections exists in the Netherlands, where the Labour Act which protects employees against health and safety hazards associated with performing paid labor covers self-employed individuals if they are hired by an employer, such as a cleaner hired by a school.

Employment classification is a fast-moving landscape and various countries have engaged in court cases that contest the classification of digital platform workers as self-employed.^41^ In Canada, unions and digital platform workers won a court decision in 2020 that Foodora digital platform workers were “dependent contractors” eligible to form a union, which led to Foodora promptly closing its Canadian operations.^42^ However, Foodora has recognized itself as an employer in some European countries (e.g., Norway, Sweden, Denmark) and continues to operate in these jurisdictions.^43^ It is possible that platforms’ attempts to misclassify workers were tempered by strong traditions of labor protection and collective organization in Nordic countries.^44^ However, Denmark had a similar experience to Foodora in Canada when Uber chose to withdraw from the country in 2017 following a new political agreement on the Taxi Act. In this case, when Uber first came to Denmark in 2014, the Danish Transport Authority reported Uber to the police on the grounds that they violated the Danish Passenger Transport Act by driving in non-approved cars and using non-certified drivers, and prosecutors filed charges against Uber, resulting in Uber closing operations in Denmark.^45,46^ Further, in 2022, Deliveroo in France received a substantial fine for not declaring their drivers as employees.^47^

Other changes are under way. In New Zealand, the Employment Court found in a 2020 case that Uber drivers should be regarded as self-employed contractors, not employees; however, trade unions are preparing to challenge that ruling.^48^ In Finland, the Labour Council ruled in 2020 that food couriers should be considered as employees; however, there have since been several more mixed judgments by other Finnish authorities. As well, in September 2021, the Netherlands Amsterdam district court ruled that Uber drivers were employees, not freelancers^49^ but Uber has indicated plans to appeal this ruling. In France, the election of a regulatory agency for digital platforms is under way.^50^ This suggests that the status and social protection of digital platform workers will be dealt with through national-level collective bargaining. As a result of employment-related legal judgments and to move away from piece-by-piece court rulings, the classification of digital platform workers in Europe may change following a December 2021 EU wide proposal of presumption of employment.^38^

Misclassification of workers has implications for taxation, for both workers and digital platforms. For instance, in the Netherlands, self-employed digital platform workers are required to pay income tax but it is difficult to identify the official employer. Criticism in the Netherlands has centered on the unequal playing field for platform workers compared to regular employers.^51^

A complication regarding reform of employment classification is that different legal acts categorize workers differently. This complexity is illustrated by the case of Denmark where labor inspection services, the workers’ compensation board, and also tax authorities each use different criteria for determining whether a person working on a platform is considered to be employee (or solo-self-employed) and thus eligible, for instance, for workers’ compensation and coverage by the Working Environment Act. Each law has different criteria, some of which are presently in court case trials. A further example is Ontario where temporary agency workers are considered employees of the agency under the Workplace Safety and Insurance Act but under the Occupational Health and Safety Act, the “client employer” and the agency are held jointly responsible.^52^ Misclassification also has implications for digital platforms. Classifying workers as self-employed largely enables platform companies to avoid paying payroll taxes, including employment insurance and pension plans. They also avoid other employee costs, such as vacation pay and parental leave. Indeed, a recent estimate found that Finnish companies save approximately 34% when social insurance costs are shifted to the contractor or self-employed person.^53^

In all, given current challenges with employment misclassification of platform and other workers, it is clear that refined criteria are needed to establish employment status. In the following sections, we examine how self-employed and low-income workers are protected by basic employment and safety standards. We also consider their access to unemployment and sickness or injury benefits.

2. *Basic employment and safety standards*

Minimum wage and occupational health and safety standards are key provisions in most advanced economies and are intended to ensure a decent and safe work environment. In this section, we depict the changing landscape of how these standards protect precariously employed workers.

*Minimum wage*. The income security of workers is often protected by minimum or collectively negotiated wages. Minimum wages, which generally appeared first in countries with high inequality rates and low collective bargaining coverage, have been found to reduce income inequality.^54,55^ However, some of our jurisdictions (Denmark, Finland, Sweden) have no minimum wage. These countries instead have a high proportion of unionized workers and their collective bargaining with employer organizations results in decent wages for most workers.

Minimum wage standards appear to provide income protection to workers if they have full-time working hours. Although minimum wage rates have risen in some countries, such as New Zealand, low-wage employment is persistent in liberal states.^56^ In our sample, Canada and France are examples of countries where workers' incomes remain below the poverty line despite receiving the minimum wage.^56,57^ This is troubling because the number of minimum wage earners in Canada doubled between 1998 and 2018.^58^ A further challenge is that minimum wage is sometimes defined per hour or day,^59^ and there are substantial populations of working poor who have only on-call or part-time working hours. For instance, it is estimated that 220,000 (2.4%) workers in the Netherlands are poor because of insufficient or irregular work.^60^

Some jurisdictions have started to require a minimum wage for digital platform drivers, but outside of an “employee” employment classification. The Uber drivers designated as “workers” in the UK are entitled to a minimum wage and vacation time. In 2022, Canada's largest province of Ontario passed the “Digital Platform Workers Rights Act” for (self-employed) digital platform couriers that enforces minimum wages.^61^ However, it has been criticized for limiting minimum wages to “active time” on the app, which excludes waiting times.^62^

In June 2022, the EU provisionally approved a directive on a minimum wage or similar arrangement for workers who currently lack sufficient wage protection.^63,64^ EU countries with minimum wages will need to ensure that these wages are adequate compared with average incomes. Adequate wages are considered to be those that ensure a decent living for workers, strengthen incentives to work, and reduce in-work poverty and inequality at the lower end of the wage distribution.^64^ Based on the observation that the majority of EU countries with high levels of minimum wages relative to the median wage have a collective bargaining coverage above 70%, the directive requires that countries without a minimum wage have at least 70% of workers covered by collective agreement. States that do not reach this threshold level are required to provide for a framework for collective bargaining and establish an action plan to promote collective bargaining.^64^ Given growing numbers of “working poor” people in Europe^65^ and internationally,^66^ we see this directive as a step in the right direction for worker well-being.

*Occupational health and safety standards.* In our eight countries, laws require employers to assess risks at work and to take appropriate measures to eliminate or reduce those risks. Further, in the Nordic countries, workers have a say in conditions through the Nordic Labour Market model, which constitutes a long tradition of cooperation between social partners and the state, including collective agreements and participative approaches.^67^ Without these measures for oversight and obligation, self-employed workers are responsible for both legal and financial aspects of their own health and safety. For instance, in Ontario, Canada, employees have a right to refuse unsafe work.^10^ However, these laws are difficult to apply to self-employed workers as they are considered to be their own boss. In these legal environments, digital platforms have borne no responsibility for the health and safety of workers, even when algorithms push them to work long hours and take on risky situations.^68,69^ Essentially, workers are made responsible for their own occupational health and safety but are not provided with tools or supports to make safe decisions about work. As well, low-wage workers in general (including self-employment platform workers) face difficulties accessing their rights because of uneven power relationships between employers and workers. That is, precariously employed workers have been found to be fearful about “speaking up” about unsafe work environments for fear of overt (e.g., dismissal) or covert reprisals (e.g., given fewer/worse shifts).^11,12^

In New Zealand, self-employed workers (including digital platform workers) are additionally held responsible for the health and safety of their customers as they have obligations as “employers” under the Health and Safety at Work Act 2015.^70^ The act is aimed at protecting the health and safety of all people, including customers, in workplaces and places obligations to ensure this, including penalties for failing to do so, on “persons conducting a business or undertaking,” which includes the self-employed. Ironically, even though the act aims to protect the health and safety of all people, not only does it deny protection to platform workers as employees, it defines “customer” in such a way that there would be little or no possibility that a platform worker could be deemed a customer of the platform.

The European Agency for Safety and Health at Work asserts that current legislation is not aligned with conditions of the platform economy and suggests that the vague categorizations of employers and employees on platforms complicate the placement of occupational health and safety responsibilities.^71^ For instance, the ability of a passenger to grant or withhold favorable ratings that can affect the driver's future work prospects, puts drivers in a position of acquiescing to unsafe passenger demands (such as speeding or putting too many people in a car) for fear of a negative rating.^69^ Ultimately, work organization of platform companies creates a situation where no one party clearly takes responsibility. Furthermore, the legislation varies across national borders; therefore, workers fall into different categories across member states of the EU and employment relationships have often been established through the courts on a case-by-case assessment or via legal precedents.^72^ However, changes are expected following the December 2021 European Commission proposed directive that proposes evaluating the risks of automated monitoring and decision-making systems in relation to the safety and health of platform and ensuring that safeguards are in place.^38^

In the above-mentioned situations, we have considered proposed standards to clarify employment status, which in turn will protect the health of workers. We have also considered existing standards and their deficiencies. In the next section, we address benefits in place to support workers in the event of unemployment or illness/injury.

3. *Key worker protections and benefits*

Social security support for people without income because they lack employment or cannot work due to injury or illness is a key aspect of our social security systems. However, these supports often do not apply to precariously employed workers.

*Unemployment benefits.* When workers are unable to find work, most systems provide unemployment benefits to support the workers during their transition to next job. Such benefits often also extend to caregiver leave and short-term injury or illness. In most of our jurisdictions, unemployment benefits are limited to employees and contingent on previous hours worked. For people excluded from unemployment benefits, most systems also have basic social assistance for people regardless of former employment status. Unlike other countries discussed in this commentary, New Zealand does not have a system of unemployment benefits linked to previous employment or social insurance contributions. Instead, all individuals irrespective of work history are directed to the basic social assistance system for tax-funded, flat-rate benefit if they are unemployed or seeking work. This benefit is relatively low (about 30% of after-tax average wage for a single person) and is means tested against joint spousal income, so that a person with an employed partner will typically not qualify for assistance.

In Canada, self-employed workers have the option to pay into employment insurance benefits for parental leave, caregiver leave, and short-term injury or illness and in Germany they can opt into unemployment insurance; however, it is unclear how many self-employed people actually take up these opportunities. They may be unaware of this support or lack the income to afford this coverage.

Example of rules about previous hours worked include Denmark, where unemployment benefits are not available to employees who work 8 h or less each week and the Netherlands where 26 weeks of work are needed.^73^ Similarly, in Ontario, Canada, 420 h of insurable earnings within the last year are required to be eligible for Employment Insurance, and only if workers were laid off (i.e., workers are ineligible if they voluntarily leave the job). These types of rules put part-time and variable-hours contract workers at a disadvantage as they may not accumulate the necessary hours to be eligible for support. They usually exclude the self-employed, except in our sample in Germany where the self-employed can pay to opt in. However, even if self-employed platform workers were included in schemes, their uneven work hours would likely exclude them based on minimum hours worked policies. These exclusions mean that, in a country such as Denmark with its “flexicurity” system, 10% of the work force is excluded from the security and the protection that applies to the majority of Danish employees.

*Sickness and injury benefits.* When ill or injured, most workers in advanced economies have access to sickness and injury benefits that provide some degree of income replacement and/or healthcare coverage. For instance, in Sweden, Finland, and Canada, all residents have universal access to basic healthcare and health-related social insurance, regardless of their employment status. However, a key distinguishing feature among these countries is the generosity of income support benefits paid.

In Nordic countries, the amount is economically sustainable for the applicant or even higher than that earned with unemployment benefits. In contrast, Canada income support for non-work-related permanent illness and injury is provided through basic welfare payments that leave recipients with incomes below the poverty line. In France, although injured or ill workers receive financial compensation for physical injury, this compensation has been criticized for being insufficient due to its lump-sum nature.

Separate systems for income support for injuries and illnesses that can be traced to work were common across our countries, but play an especially prominent role in a country like Canada where income support benefit levels for non-work-related injury/illness are very low but income support to the work-injured/accident victims is similar in generosity to disability benefits provided in Nordic countries, and calculated at a high percentage of former wages, up to a cap. In Canada, this uneven support has colloquially been called the bicycle (welfare) versus the Cadillac (workers’ compensation) support model. However, there are some exclusions. In Canada, not all sectors are covered by workers’ compensation; for instance, in Ontario, only 70% of businesses are required to be covered.^74^ A unique model is New Zealand, where the Accident Compensation scheme covers all work and non-work accidents and provides a high percentage of prior earnings, up to a cap, where the injury results in income loss due to an incapacity to work. However, less generous support exists for those with long-term illness, who must turn to basic welfare payments.^75^

In Ontario, Germany, and the Netherlands, self-employed workers can “opt in” to workers’ compensation coverage by paying premiums. However, these premiums are costly and digital platform workers are not known to do this as their earnings are low. As well, in the case of the Netherlands, the coverage is only accessible if workers opt in within 13 weeks of moving from employee status to self-employed. In Ontario, exceptions exist where self-employed construction workers are required to opt into workers’ compensation and free coverage is provided to foot and bicycle couriers, including digital platform workers.^76^ In France, self-employed farmers are required to have workers’ compensation coverage and other self-employed workers can voluntarily opt-in for this coverage.^77^ However, as of 2016, France required digital platforms to cover accident insurance costs of self-employed platform workers.^78^

Across our countries, return-to-work programs exist for employees who are ill or injured. These provide income support and health accommodations to support work reintegration. However, the coverage of these programs is limited; they generally exclude self-employed workers and precariously employed workers can face difficulty accessing them. For instance, in Sweden and the Netherlands, workers in precarious employment arrangements, such as call-in-workers, often do not have an employment contract with fixed hours and their employers may not fulfil return-to-work obligations.^79^ As well, when low-wage workers have two part-time jobs, they may be left unsupported due to lack of clarity about who is responsible for the support. This is the case in Sweden, where employers pay the wages of injured or ill workers for the first 14 days.^80^

4. *Protections created to support workers during the pandemic*

Measures undertaken by different jurisdictions during the COVID-19 pandemic lockdowns provide a glimpse of possible improvements to social safety nets for low-wage and self-employed workers. Many jurisdictions added benefits and relaxed access to entitlements for employees. For instance, Finland provided social insurance benefits during formal isolation and quarantine periods, although these benefits were paid to the employer and only for formal measures.^81^ Denmark relaxed job search requirements for those on sickness benefits.^82^ Most countries provided support to companies in hard hit sectors, such as restaurants, to retain employees even when operations could not be maintained.

Examples of a slightly more inclusive system during the pandemic include Sweden and Canada, where unemployment and sickness benefits entitlement criteria were somewhat relaxed for precariously employed workers. In Sweden, the state covered the costs for waiting days for both the employed and the self-employed.^83^ In some Canadian provinces, precariously employed workers, who normally have no paid sick days, gained a few paid sick days as a temporary measure, with government reimbursing employers.^84,85^

In most of our jurisdictions, income support coverage during COVID-19 (up to early Wave 4 in September 2021) was adapted to provide coverage to self-employed people. For instance, the Netherlands provided income replacement for self-employed who were earning less than the minimum wage level. In Denmark, measures were introduced to support solo self-employed workers, freelancers, and entrepreneurs if they could document that they had lost up to 30% of their revenue due to the pandemic.^86^ In Canada, Finland, and Germany, self-employed workers temporarily received income support benefits.

Did these policy changes make a difference to the health of populations in the countries addressed? It is difficult to tell. While literature is emerging about effects of COVID-19 measures, impact studies are still at early stages (e.g., focused on the need for coordination of actors^87,88^) and population health outcome data are not yet available as linked to specific measures.

## Discussion

In 2019, the OECD's report on the future of work noted that social protection provisions need to be reshaped to ensure better coverage of workers in non-standard forms of employment.^22^ Two years into a global COVID-19 pandemic, our societies are in a fluid situation, with social values that may be shifting as the pandemic renews our views on social interdependence. The risks faced by many low-wage and digital platform workers who kept food and other essential services running are becoming recognized in the public imagination. Advanced economy countries are arguably at a critical juncture, ripe for re-thinking social contracts and engaging in policy change.

Our analysis reflects on how, to date, few laws have protected the income stability and health of low-wage workers in our countries, including digital platform workers, although during the COVID-19 pandemic it became clear that social security access rules could be altered to better support low-wage workers, including self-employed digital platform workers. Across all eight countries, digital platform workers have been largely recognized as only self-employed. Ongoing court battles in various jurisdictions may change this employment recognition, but a complication is that definitions of “who is an employee” usually vary across legislative acts, meaning that wholescale legal changes across jurisdictions are needed. A critical recent development is the December 2021 European Commission proposed directive to improve the working conditions of people working through digital labor platforms by referring to the platform as the employer who has the responsibility for the working conditions. The proposed directive, which serves as an international role model, aims to clarify employment status and increase transparency regarding algorithmic management. In our view, this is a positive development for both workers and employers. If passed, it could bring many workers into the protection of labor laws and social security coverage, and will level the competitive playing field for other employers vis a vis platform employers.^89,90^ As of December 2021, there were more than 100 court decisions and 15 administrative decisions in the EU dealing with the employment status of people working through platforms.^89^ If implemented, this directive should lead to fewer court battles over employment status and strengthen collective bargaining opportunities for workers. However, the directive will be exposed to lobbying and may become watered down during the political process.^91,92^

A key issue pertinent to low-wage workers is whether a minimum wage supports a decent living wage. That is, do minimum wage standards serve to legitimate a deregulated labor market or do they actually promote decent living wages?^56^ The issue of living in poverty despite working full-time is a topic of political concern in countries such as Canada^93^ and in France.^94^ A further issue is workers who can access only irregular or part-time work and so live in poverty even in the presence of a minimum wage. In the case of the Netherlands, the working poor are mostly composed of workers with inadequate work hours, and they are often ineligible for social assistance.^60^

A recent draft EU directive focuses on the issue of adequate minimum wages.^64^ While this directive optimistically notes that a minimum wage may address some of the risks faced by platform workers, it is important to reflect that this is only if the platform workers are legally considered as employees.

Protection against unsafe working conditions via occupational health and safety standards is a particularly pertinent issue for self-employed digital platform workers, as platforms can push workers to engage in risky work via algorithmic prompts and punishments, particularly during the pandemic (e.g., accepting unmasked riders to ensure good rider ratings).^95^ The non-transparency of algorithms and lack of recourse for platform workers to resist unfair conditions has been a much-needed area for policy development. Protection is also needed in jurisdictions, such as New Zealand, where, despite a notable lack of control over their working conditions, laws hold self-employed platform workers responsible for the health of their customers. Further, attention is needed where, despite the existence of protective policies, there is a lack of enforcement by labor inspectorates, prosecutors, and courts.

Emergency measures put in place by governments during the pandemic demonstrate that protections for low-wage and digital platform workers are possible.^83^ It was instructive to see how some jurisdictions could quickly relax rules during lockdowns; for instance, allowing self-employed workers and workers with few working hours to access income replacement funds. These measures also illuminate problems with systems that deliver supports to workers via employers as this pathway does not reliably reach workers on precarious employment contracts or the self-employed.

Our consideration of work-related social security protections across eight countries provides insight into varying types of support for low-wage and self-employed workers. Solutions to better support self-employed digital platform and low-wage workers are varied and, broadly speaking, have centered on three approaches. The first is to strengthen employer obligations: this is where we see proposals for minimum wages and court arguments about misclassification of digital platform workers. This is the focus of the European directive to improve the working conditions of people working through digital labor platforms. A second approach is to strengthen the social security net so that, even with poor employer support, workers can gain access to well-being and a decent life. The latter approach centers on approaches such as basic income schemes that provide a basic income to anyone unemployed or working with an income below a certain income level.^96,97^ Digital platform companies, such as Uber, have pushed for a “third way” that would continue to relieve them of employer responsibilities (e.g., not paying into employment insurance and national pension schemes) but that includes organizing some sort of benefits pool for their workers.^98^ For instance, Uber's “Flexible Work + ” proposal was a key part of the conservative platform during the 2021 Canadian federal election.^99^ This approach presently exists in the UK, where London Uber drivers were recently determined to be a hybrid, called “worker” rather than a full “employee.”^29^ It is also present in France, where platforms are required to cover accident insurance costs of self-employed workers^78^ and in Ontario, Canada where platforms are required to pay self-employed workers a minimum wage for “active” platform time.^61^ However, we consider “third way” approaches as unfair to workers and society because they create an unfair competitive playing field for platform companies vis a vis regular employers who are required to assume costs of payroll taxes^100^ and employment standards. As well, when platform workers are ill, injured, or under-employed, it is generally taxpayer-funded national welfare systems that support workers and pick up the costs in lieu of platform companies.

Our commentary raises questions, many of which have been asked before.^15,16,101,102^ What are the advantages of expanding the definition of employers, including employer responsibilities, versus expanding the eligibility of universal protection by the state, e.g., basic income? An example of expanded employer responsibility is Australia's 2019 Fair Work Act,^103^ which holds end user businesses responsible for deficiencies in the management of labor supply chains. With this accessorial liability, employers at the top of supply chains are liable for contraventions by sub-contractors for issues such as fair pay and safe working conditions.^104,105^ This has a positive effect on employment standards.^105^ However, even with fair wages and decent working conditions precarious employment is not evaded as workers may have low incomes due to few working hours. As such, we advocate for both expanded employer responsibility and more secure basic incomes.

Other questions include: How can laws be reformed so that all workers have equal right to occupational health protections and services? How can definitions of employees and employers become better aligned across varied employment and occupational health laws? How can solidarity between “have” and “have not” working groups be promoted?^106^

Overall, our review shows a persisting “haves” and a “haves not” divide, in line with “insider-outsider” divides described by Emmenegger et al.^107^ and the polarized job environment described by Kalleberg.^1^ We have briefly described how workers in traditional jobs have been provided with fairly good social protection for work and health, which has been especially apparent during the ongoing COVID-19 pandemic. We focused on the other end, where low-wage and self-employed digital platform workers made small and often temporary gains during the pandemic in some jurisdictions. Mostly, they have been left out of schemes, despite International Labour Organisation principles about decent work.^15^ In the context of an evolving social contract during the COVID-19 pandemic, this paper provides views on avenues for policy reform and research from employment and occupational health specialists across eight advanced economy countries.
